# Elastic Fibre Prestressing Mechanics within a Polymeric Matrix Composite

**DOI:** 10.3390/polym15020431

**Published:** 2023-01-13

**Authors:** Hui Chen, Folian Yu, Bing Wang, Chenmin Zhao, Xiayu Chen, Walter Nsengiyumva, Shuncong Zhong

**Affiliations:** Fujian Provincial Key Laboratory of Terahertz Functional Devices and Intelligent Sensing, School of Mechanical Engineering and Automation, Fuzhou University, Fuzhou 350108, China

**Keywords:** carbon-fibre-reinforced polymeric composite, prestress, elasticity and viscoelasticity, fibre/matrix interface, in-plane stress, mechanics

## Abstract

The elastic fibre prestressing (EFP) technique has been developed to balance the thermal residual stress generated during curing of a polymeric composite. The continuous fibre reinforcements are prestressed and then impregnated into a polymeric matrix, where the prestress load is only removed after the resin is fully cured in order to produce an elastically prestressed polymeric matrix composite (EPPMC). Although the EFP is active in improving the static mechanical performance of a composite, its mechanics on dynamic mechanical performance and viscoelasticity of a composite is still limited. Here, we established a theoretical model in order to decouple the EFP principle, aiming to better analyse the underlying mechanics. A bespoke fibre prestressing rig was then developed to apply tension on a unidirectional carbon-fibre-reinforced epoxy prepreg to produce EPPMC samples with various EFP levels. The effects of EFP were then investigated by carrying out both static and dynamic mechanical testing, as well as the viscoelastic creep performance. It was found that there is an optimal level of EFP in order to maximise the prestress benefits, whilst the EFP is detrimental to the fibre/matrix interface. The EFP mechanisms are then proposed based on these observations to reveal the in-plane stress evolutions within a polymeric composite.

## 1. Introduction

Polymeric matrix composites (PMC) are superior in terms of specific strength, specific modulus, corrosion resistance, and designability. They have been widely used in industries, such as automotive, tissue engineering, aerospace, and sustainable engineering [1,2]. Manufacturing of traditional PMC products usually undergoes heating, pressurisation, cooling, and curing processes. Owing to mismatch of the thermal expansion coefficient between fibre and matrix materials, thermal residual stress commonly occurs within a composite structure [3]. Although accumulation of residual stresses can occasionally be beneficial, especially for producing shape-changing bistable or multistable structures [4,5,6,7,8], they are usually detrimental, which may lead to laminate warping, buckling or micro-cracks, which significantly affects the mechanical performance of composite products [9]. PMCs suffer from breakages of fibre bundles and observations of cracks [10] due to the exerted large interfacial forces with surfaces coated with cellulose nanocrystals [11], whose rod-shaped morphology treasures high-aspect ratios, which is common in the case of the studied carbon fibre composites. These studies evidence the necessity to conduct further research in this field.

There have been many techniques to reduce the generated thermal residual stress within a composite, including heat treatment after the curing process [12]; regulating curing cycles [13,14]; addition of various types of nanoscale particles to reduce any mismatch in mechanical properties of the constituents [15,16]; physical and chemical modifications of the constituents [17]; exploiting partial relaxation of residual stresses due to viscoelasticity [18]; developing low temperature curing resins [19], as well as fibre prestressing techniques [20,21]. Among these, fibre prestressing was first developed in the 1960s, which is able to reduce or eliminate residual stresses from the manufacturing of composites, without changing their composition, increasing mass or product dimensions [22].

Depending on the mechanical properties of the fibre materials, fibre prestressing can be applied on both elastic fibres and viscoelastic fibres to produce (i) an elastically prestressed polymeric matrix composite (EPPMC) and (ii) a viscoelastically prestressed polymeric matrix composite (VPPMC) [23,24]. For (i), EPPMC is produced by applying tension on fibres, which is maintained throughout the curing process. It is suitable for fibre materials with significant elastic performance, such as carbon fibre [25], glass fibre [26,27], etc. Whilst for (ii), tension is applied on viscoelastic fibres which undergo creep or stress relaxation, then the tensile load is released and the prestressed fibres are cured into a matrix. The suitable fibres mainly include semi-crystalline polymeric fibre materials with significant viscoelastic effects [28]. Because elastic fibre-based composites have been mainstream and widely applied in industries, we have focused on investigating further the elastic fibre prestressing (EFP) technique.

The EFP was first applied on polymeric composites by Zhigun [26], and later by Tuttle [29], where the prestressing concept was derived from prestressed concrete. As a well-known concept, prestressed concrete is produced by applying tension to steel rods and then embedding in liquid concrete. The tension is maintained during the solidification of the concrete and then released when fully solidified; thus, the elastic deformation of the steel rods is locked inside the concrete. Owing to the attempted contraction generated from the elastic recovery of the rods, a compressive prestress is generated within the concrete matrix and corresponding tensile prestress within the rods, and hence the overall mechanical properties of the reinforced concrete can be improved [30]. To date, it has also been demonstrated that the EFP technique offers mechanical benefits for continuous-fibre-reinforced PMCs.

For tensile properties of EPPMCs, Brown et al. [31] found that the tensile strength of a unidirectional graphite fibre epoxy composite was increased by 17% after elastic prestressing, whilst the tensile modulus was not affected. For unidirectional glass-fibre-reinforced polymer (GFRP), Hadi [32] et al. prestressed glass fibre by winding the fibre on a drum and studied the influences of prestress level (from 0 to 200 MPa) and fibre volume fraction (*V*_f_) (35%, 45%, and 50%). It was found that the elastic modulus and tensile strength of GFRP are proportional to the prestress level and fibre volume fraction. Scherf [33] prestressed a single bundle of carbon-fibre-reinforced PMC by hanging a heavy object at the end of the fibre and found that fibre prestressing could increase the shear strength and fracture segments of the composite, resulting in a decrease in effective length and tensile modulus of the fibres. Hassan et al. [34] improved the tensile properties of unidirectional carbon-fibre-reinforced polymer (CFRP) by changing the fibre prestress level (up to 80 MPa) and the curing temperature of the composite (ambient, 80, 115, and 150 °C). They both affect the EFP effectiveness when cured at 115 °C. The tensile modulus can be increased by 85.1% under a prestress level of 20 MPa.

EFP can also be applied to improve the impact resistance of PMCs [35]. Motahhari et al. [36] conducted Charpy impact tests on prestressed unidirectional GFRP with 40% *V*_f_ and found that there is an optimal prestress level; the impact strength is maximised at a prestress level of 60 MPa. Nishi et al. [37] studied the influence of prestress level (up to 17.6 MPa) on the impact resistance of unidirectional CFRP with a fibre volume fraction of 50%, and they found that under the same curing condition, the impact strength could be improved by 30% at a prestressed level of 17.6 MPa.

For flexural performance, Motahhari et al. [38] applied EFP to unidirectional GFRP with 40% *V*_f_, and the bending strength and modulus were increased by 33%. It was found that the increase in curing temperature also changed the optimal prestress level. Zaidi et al. [39] used a screw tensioning frame to apply constant prestress strain to flax fibres. The flexural strength and flexural modulus were increased by 34% and 26%, respectively, at a prestrain level of 0.03 and 50% *V*_f_.

Therefore, research into EPPMC mainly focuses on limited aspects of static mechanical analysis, and the prestressing mechanisms are derived based on restricted observations. There is a lack of systematic and comprehensive investigation into the EFP effects on PMCs, especially on their dynamic thermomechanical properties, as well as the viscoelastic creep performance. Here, we established a theoretical model to decouple the EFP principle, aiming to better analyse the underlying mechanics. A bespoke EFP rig was then used to apply EFP on a unidirectional (UD) carbon-fibre-reinforced epoxy prepreg to produce EPPMC samples with various fibre prestressing levels. Effects of prestress level were then investigated by carrying out testing in terms of tensile, impact, creep, as well as dynamic mechanical analysis (DMA) on the EPPMC samples. The EFP mechanisms were then proposed and discussed to reveal the in-plane stress evolution within the composite.

## 2. Theory of Elastic Fibre Prestressing

**Figure 1:**
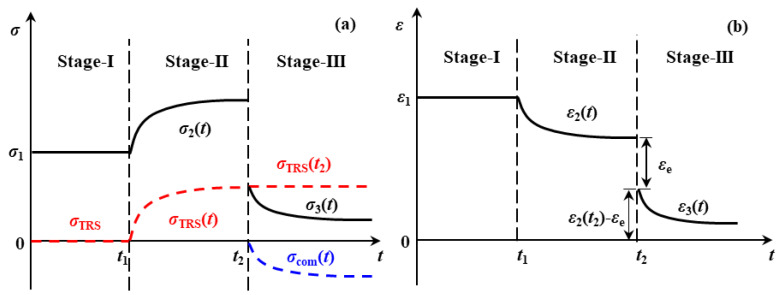
Schematic of production principle of EPPMC showing the (**a**) stress and (**b**) strain evolutions at three stages, where Stage-I is the fibre prestressing stage; Stage-II is the curing of matrix; and Stage-III is the post-processing stage. The continuous black line shows the in-plane (**a**) stress and (**b**) strain evolutions during production of an EPPMC. The dashed red/blue lines show the evolution of thermal residual stress/compressive stress within a composite.

To produce an EPPMC, elastic fibres are prestressed at a designated level and impregnated in an uncured resin. The tensile load is released only after the matrix is fully cured. Thus, the preparation of an EPPMC using unidirectional carbon prepreg can be divided into three stages, see Figure 1. The stress and strains carried by the fibre and composite are as follows:

Stage-I is the fibre prestressing stage, where constant tensile stress, $\sigma_{1}$, see Figure 1a, is applied on unidirectional fibres, leading to a constant strain, $\varepsilon_{1}$, see Figure 1b. The stress and strain carried by the uncured prepreg are proportional in the elastic deformation region and follow Hooke’s Law as presented in Equation (1) below:

(1) $$\sigma_{1}{=E}_{\mathrm{pre}}\varepsilon_{1}$$

where, $E_{\mathrm{pre}}$ is the elastic modulus of an uncured fibre prepreg.

Stage-II is the curing stage of EPPMC. The fibre prepreg in the pre-tensioned state is cured under constant stress, $\sigma_{1}$, corresponding to $t_{1}$~$t_{2}$ in Figure 1a. Owing to the mismatch of thermal expansion coefficient between fibre and matrix material, thermal residual stress, $\sigma_{\mathrm{TRS}}\left(t\right)$, represented by Equation (2), is generated and accumulated during Stage-II [40] and

(2) $$\sigma_{\mathrm{TRS}}{(t)=\alpha }_{c}\;\Delta {T\;E}_{\mathrm{pre}}\left(t\right)$$

(3) $${\;\alpha }_{c}=\left(\alpha_{f}E_{f}V_{f}{+\alpha }_{m}E_{m}\right)/\left(E_{f}V_{f}{+E}_{m}V_{m}\right)V_{m}$$

Here, $\alpha_{c}$ is the thermal expansion coefficient of a composite along the fibre direction; ΔT is the temperature difference; $E_{\mathrm{pre}}\left(t\right)$ is the elastic modulus of the curing fibre prepreg at time t, which increases during the curing process; $\alpha_{f}$ and $\alpha_{m}$ are the thermal expansion coefficients of fibre and matrix, respectively; $E_{f}$ and $E_{m}$ are the elastic moduli of fibre and matrix materials respectively; and $V_{f}$ and $V_{m}$ are the volume fractions of fibre and matrix materials.

Now the internal stress level within the cured composite, $\sigma_{2}\left(t\right)$, follows:

(4) $$\sigma_{2}{(t)=\sigma }_{1\;}{+\sigma }_{\mathrm{TRS}}\left(t\right)$$

Because the tensile modulus of EPPMC increases during curing, the strain carried by EPPMC is as follows:

(5) $$\varepsilon_{2}(t)=\left[\sigma_{1\;}{+\sigma }_{\mathrm{TRS}}\left(t\right)\right]{/E}_{\mathrm{pre}}\left(t\right)$$

Stage-III is the post-processing stage. On tensile creep stress removal at $t_{2}$, the elastic fibres instantly recover by a strain value of $\varepsilon_{e}$; thus, the elastic strain locked within an EPPMC is $\varepsilon_{2}{(t}_{2}{)\;-\;\varepsilon }_{e}$, assuming the stress carried by the fibres before load removal is $\sigma_{\mathrm{fpr}}^{0}$, then

(6) $$\sigma_{\mathrm{fpr}}^{0}{=E}_{f}\varepsilon_{2}{(t}_{2})$$

Now we consider the interactions between fibres and matrix, assuming the stress and strain carried by both fibre and matrix are $\sigma_{\mathrm{fpr}}$, $\sigma_{\mathrm{mpr}}$, $\varepsilon_{\mathrm{fpr}}$, $\varepsilon_{\mathrm{mpr}}$, respectively:

(7) $$\varepsilon_{\mathrm{fpr}}{=\sigma }_{\mathrm{fpr}}{/E}_{f}-\varepsilon_{2}\left(t\right)$$

(8) $$\varepsilon_{\mathrm{mpr}}{=\sigma }_{\mathrm{mpr}}{/E}_{m}$$

When curing is complete, i.e., at $t_{2}$, when creep stress is removed, the strain caused by prestress recovery of fibre and matrix is equal, and the geometric equation is

(9) $$\varepsilon_{e\;}{=\varepsilon }_{\mathrm{fpr}}{=\varepsilon }_{\mathrm{mpr}}$$

(10) $$\sigma_{\mathrm{fpr}}{/E}_{f}-\varepsilon_{2}{(t}_{2}{)=\sigma }_{\mathrm{mpr}}{/E}_{m}$$

The interaction force between fibre and matrix is equal, so the static equation is

(11) $$\sigma_{\mathrm{fpr}}V_{f}{+\sigma }_{\mathrm{mpr}}V_{m}=0$$

Stress induced by fibre prestressing is, thus,

(12) $$\sigma_{\mathrm{fpr}}{=E}_{m}V_{m}E_{f}\varepsilon_{2}{(t}_{2})/\left(E_{f}V_{f}{+E}_{m}V_{m}\right)$$

(13) $$\sigma_{\mathrm{mpr}}=-E_{m}V_{f}E_{f}\varepsilon_{2}{(t}_{2})/\left(E_{f}V_{f}{+E}_{m}V_{m}\right)$$

Because the elastic recovery of the fibre is usually instant, the elastic strain locked inside an EPPMC, i.e., $\varepsilon_{2}{(t}_{2}{)\;-\;\varepsilon }_{e}$, will tend to recover and interact with the matrix. Because the polymeric matrix is a viscoelastic solid, the generated compressive stress, $\sigma_{\mathrm{com}}\left(t\right)$, grows nonlinearly with time [41]. The strain $\varepsilon_{3}\left(t\right)$ is now

(14) $$\varepsilon_{3}(t)=\left[\sigma_{\mathrm{com}}{(t)+\sigma }_{\mathrm{TRS}}{(t}_{2})\right]{/E}_{\mathrm{EPPMC}}$$

Therefore, the internal stress level within an EPPMC, $\sigma_{3}\left(t\right)$, is

(15) $$\sigma_{3}{(t)=\sigma }_{\mathrm{com}}{(t)+\sigma }_{\mathrm{TRS}}{(t}_{2})$$

Therefore, the internal stress level of a composite can be regulated by adjusting the elastic fibre prestressing level.

## 3. Experimental Design

### 3.1. Materials

Table 1 shows the mechanical properties of a UD carbon prepreg, which was supplied by Weihai Guangwei Composites Co., Ltd., Weihai, China.

### 3.2. Fibre Prestressing Rig

**Figure 2:**
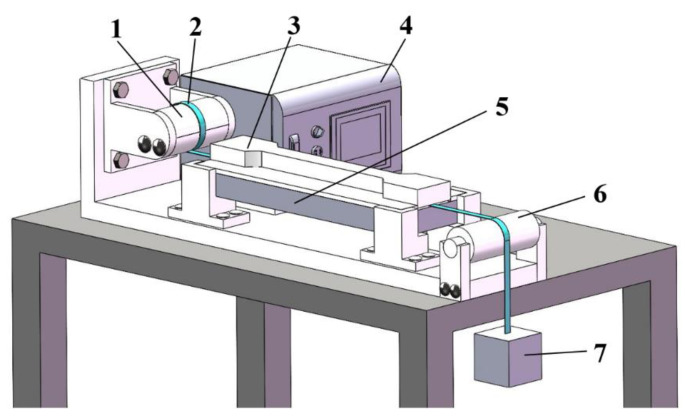
A bespoke creep stress-based fibre prestressing rig showing (1) fibre fixing clamp; (2) unidirectional fibre prepreg material; (3) upper mould; (4) and (5) heating module with a temperature accuracy of ±1 °C; (6) roller; and (7) weight platform.

According to the elastic fibre prestressing principle in Section 2, for uncured carbon prepregs, elastic fibre prestressing can be achieved by applying (i) constant strain or (ii) constant stress. For (i), fibre prestressing at a constant strain usually induces stress relaxation of the resin-saturated carbon fibres, leading to changes in fibre prestressing levels. An initial study shows that the prestress level dropped by 25% after 10 min of tension at 1% of applied strain. Whilst for (ii), it is able to maintain a constant prestress level where creep occurs on fibres. Thus, a bespoke creep stress-based fibre prestressing rig was designed and manufactured as schematically shown in Figure 2.

The fibre fixing module is composed of both upper and lower semi-cylindrical blocks where the lower half block is fixed on the base and the upper half is movable for better clamping and uniform prestressing. The heating module is composed of a temperature controller and a thermal plate with an accuracy of ±1 °C. The roller module at the end plays the role of supporting, flattening, as well as reducing the stress concentrations. The designated fibre prestressing level is achieved by adjusting the weight applied on the platform. To ensure stable and reliable fibre prestressing, the lower surface of the fixing clamp, the upper surface of the thermal plate and the upper surface of the roller, are kept on the same plane. The surfaces that may contact the fibre prepregs were all covered by mould release films.

### 3.3. EPPMC Sample Production

To produce an EPPMC, the fibre prepreg was clamped in between the fixing blocks and winded along the outer cylinder in order to be self-locking to reduce stress concentration effects. The other fibre ends were attached on the weight platform in order to be prestressed. After the creep load was applied, the thermal plate was heated at a constant rate of 25 °C/min until 80 °C, and held for 30 min, then heated to 120 °C and kept for another 90 min. The creep load was then removed and the sample was cooled to room temperature and cut into designated sizes for testing. To evaluate the effects of fibre prestressing, EPPMC samples under various fibre prestressing levels were produced, including 0 MPa, 12 MPa, 24 MPa, 48 MPa, 90 MPa, 135 MPa, 180 MPa, and 220 MPa. All samples were stored at room temperature for at least one week before testing.

### 3.4. Static Mechanical Testing

To benefit from elastic fibre prestressing, the carbon prepregs need to be prestressed within the elastic region. Thus, a universal tensile machine MTS-CMT4104 was used to apply tension on unidirectional carbon prepreg to determine the tensile performance following ASTM D3039 [42], with a constant crosshead speed of 2 mm/min and 5 kN load cell at ambient temperature. The prepreg sample was cut into 150 × 5 mm with a single layer thickness of 0.1 mm. The tensile properties of EPPMC samples produced under different prestress levels were then tested at the same conditions. The sample size was 150 × 5 × 0.2 mm, and the same prepreg was used to produce reinforced tabs to reduce stress concentrations and improve the measurement accuracy. Each test was repeated three times to evaluate repeatability.

A Charpy impact tester LX-XJJ-50 from Guangdong Aisry Instrument Technology was used for impact testing, with a pendulum impact energy of 15J. UD prestressed composite samples were tested at 25 °C following ISO 179-1:2000 [43]. Sample size was 150 × 8 × 0.4 mm, with a test span of 40 mm.

### 3.5. Dynamic Thermomechanical Testing

A dynamic thermomechanical analyser DMA1 (Mettler Toledo Group Ltd., Zurich, Switzerland) was used for three-point bending tests of the prestressed composite samples. Thermomechanical testing was performed at a heating rate of 5 °C/min until 200 °C, with an amplitude of 2 μm and frequency of 1 Hz. The sample dimensions were 40 × 5 × 0.4 mm, with a test span of 30 mm.

To evaluate the effects of prestress level on viscoelastic performance of composites, tensile creep testing was also carried out on prestressed samples at different temperatures, which include 25 °C, 50 °C, 75 °C, 100 °C, 125 °C. The sample dimensions were 20 × 8 × 0.4 mm, and the test span was 5 mm. The creep stress was set as 3 MPa, which is restricted by the DMA, and both creep and recovery were monitored for 30 min.

## 4. Results and Discussion

### 4.1. Tensile Performance

**Figure 3:**
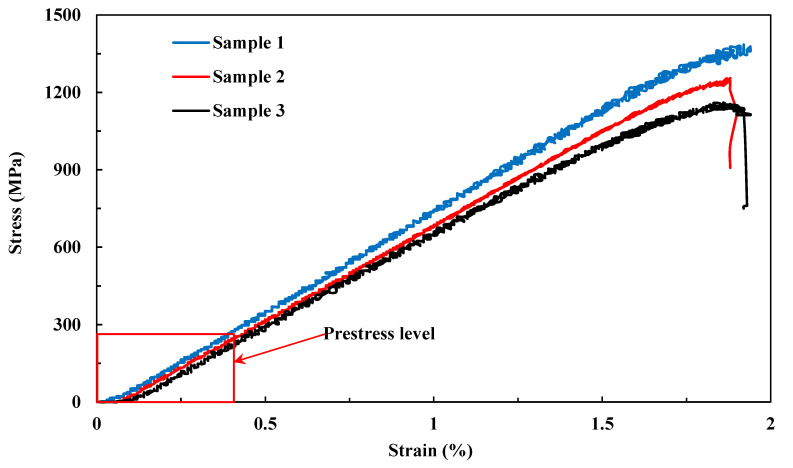
Tensile performance of the uncured UD carbon prepreg.

**Figure 4:**
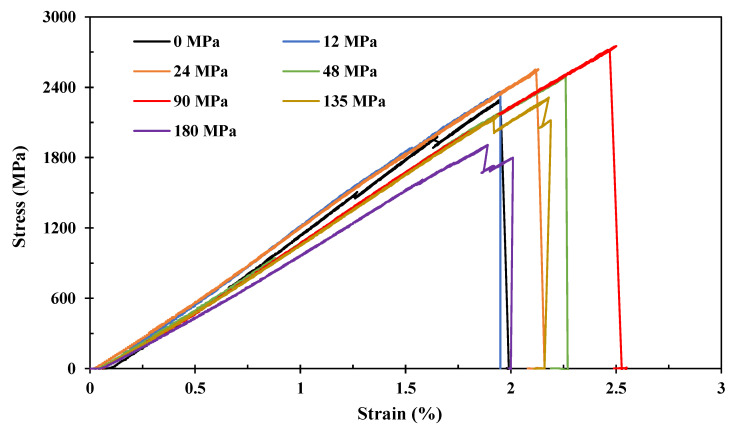
Stress–strain curves of prestressed unidirectional carbon fibre composite.

To evaluate the effects of fibre prestressing level on the tensile properties of the composite, tensile tests were first applied on the uncured UD prepreg, and Figure 3 shows the tensile performance, which was tested three times for repeatability. It also shows typical close-to-linear failure and gives an average tensile strength value of 1267.0 ± 112.9 MPa, with a modulus of 74.4 ± 4.4 GPa. Since the fibre prestressing level applied in this paper was controlled within 230 MPa, as highlighted in Figure 4, it corresponds to an elastic strain level within 0.4%.

Figure 4 shows the effect of prestress level on the tensile properties of the composite, which shows typical close-to-linear failure. It is noted that for non-prestressed composite samples, there are clear local premature failures at strains around 1.3% and 1.7%, i.e., step-like features, whereas the prestressed composite samples present straight lines until failure. This infers that fibre prestressing is able to destroy the defective fibres before being cured into a matrix in order to reduce the energy impacts from the stress waves generated by their premature failures on the adjacent fibres, which in turn improves the tensile properties of the composites.

**Figure 5:**
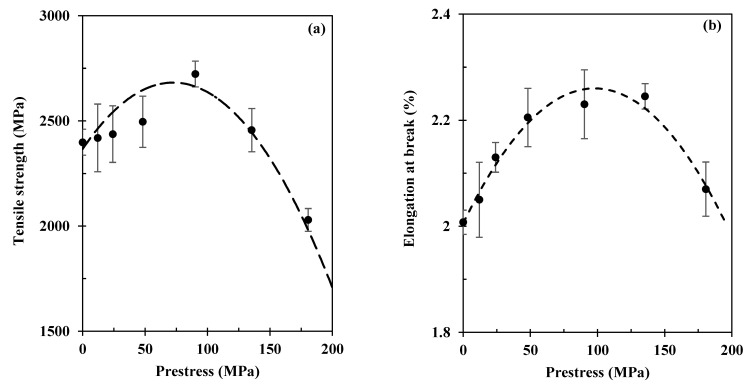
Effects of fibre prestressing level on (**a**) tensile strength and (**b**) elongation at break of the composite. Each case was tested three times, and standard errors are presented by the error bars.

Figure 5 further details the prestress effects on tensile strength and elongation at break data calculated from Figure 4. It is clear that both the tensile strength and elongation at break increases with the prestress level at the beginning. When the carbon fibres are prestressed at 90 MPa, the tensile strength is improved by 15% (i.e., 2.72 GPa compared with 2.37 GPa), and the elongation at break is improved by 11% (from 2.01% to 2.23%) when compared with as-manufactured samples. Further increasing the fibre prestress level to 135 MPa tends to decrease the fibre strength, and when 180 MPa is applied, the tensile strength becomes lower than the samples without prestress, showing that the fibre prestressing can also be harmful when over a certain limit. This indicates that there is an optimal prestress level to maximise the tensile strength. Thus, during tension, the applied tensile stress is effectively counterbalanced by the compressive stress generated by the elastic recovery, which in turn improves the tensile strength and the elongation at break.

**Figure 6:**
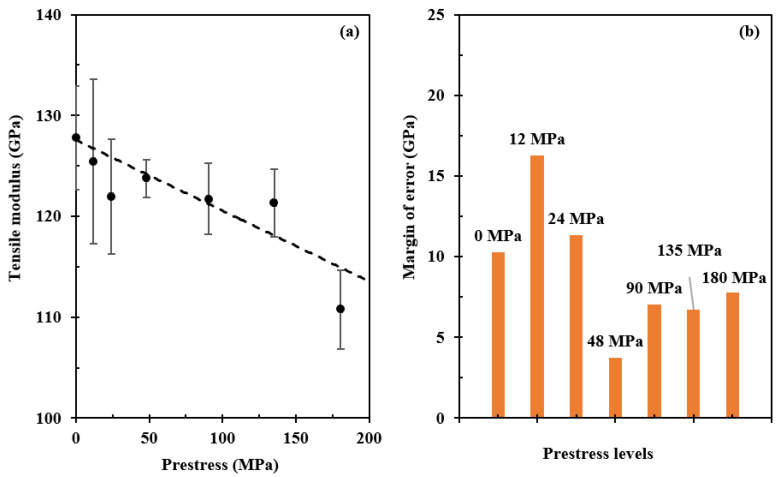
Effects of fibre prestressing level on tensile modulus of the composite. Each case was tested three times, and standard errors are presented by error bars in (**a**), and (**b**) shows the margins of error at different prestress levels marked by the data labels.

Figure 6 presents the details of prestress effects on tensile modulus calculated from the linear region in Figure 4. It shows that the modulus is negatively proportional to the prestress level, see Figure 6a, indicating that the interface strength may be influenced by the fibre prestressing. It is noted that the decrease is insignificant. There is ~14% reduction in tensile modulus when prestressed under 180 MPa compared with the non-prestressed sample. Although fibre prestressing is detrimental to the fibre/matrix interface, the margin of error decreases with prestress level and then nearly stabilises beyond 90 MPa, see Figure 6b, indicating that fibre prestressing is effective in breaking the defective fibres and increasing the straightness of the fibre bundles, which in turn increases the number of effective fibres when loaded in order to reduce the margin of errors. Therefore, as demonstrated above, both the benefits and harm from fibre prestressing need to be balanced in order to reach the maximum tensile properties.

### 4.2. Impact Performance

**Figure 7:**
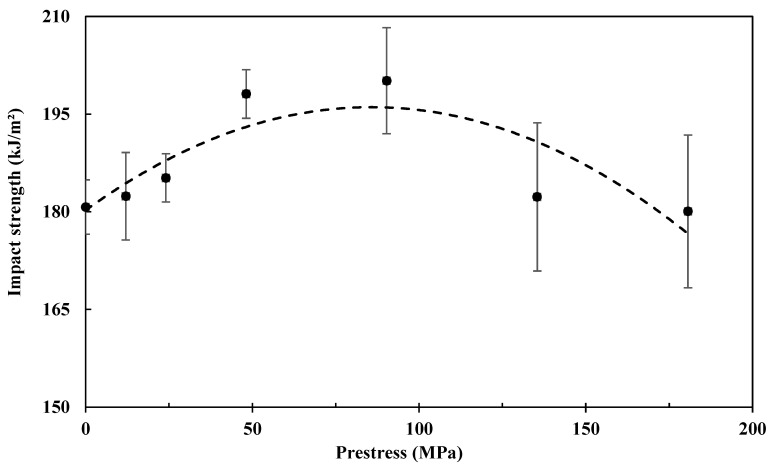
Effects of fibre prestressing level on impact strength of the composite. Each prestress level was tested six times for repeatability, and standard errors are presented by the error bars.

Figure 7 shows the prestress effects on the impact strength of the composite, together with the standard error bars. Again, it is found that there is an optimal level in terms of fibre prestress level to maximise the impact strength of the composites. In comparison with the non-prestressed composite samples, the impact strength increases with the prestress level, which is maximised at around 90 MPa of creep stress, with about 11% improvement. As stated earlier, it is known that the elastic recovery of the prestressed carbon fibres would generate compressive stresses within the composites, which further lead to residual shear stresses in the fibre/matrix interface regions. The residual shear stresses induced by fibre prestressing could promote interface debonding by absorbing more impact energy when subjected to transverse impacts. Thus, the impact strength could be improved. Further improving the prestress level may lead to internal damage to the fibres or excessive debonding of the fibre and matrix interfaces that are detrimental to impact performance.

### 4.3. Dynamic Thermomechanical Performance

**Figure 8:**
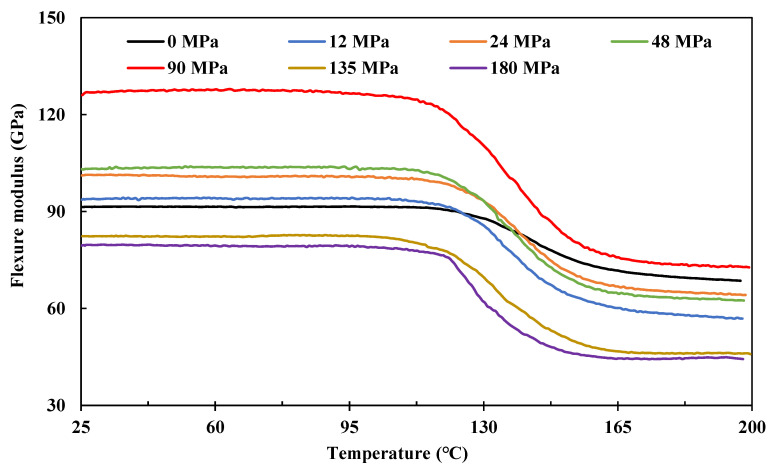
Effects of prestress level on the flexure modulus of the composite determined from three-point bending under temperature sweep from 25~200 °C.

Figure 8 shows the effects of fibre prestressing level on the flexure modulus determined from three-point bending under a temperature sweep from 25 °C to approximately 200 °C. Basically, it shows typical dynamic thermomechanical performance, clearly showing the glassy region (25~110 °C), glass transition region (110~160 °C), and the rubbery plateau region (160~200 °C). The composite also shows prestress level-dependent dynamic behaviour. When prestressed at 90 MPa, the prestress benefits are superior in terms of the flexure modulus throughout the whole temperature range.

**Figure 9:**
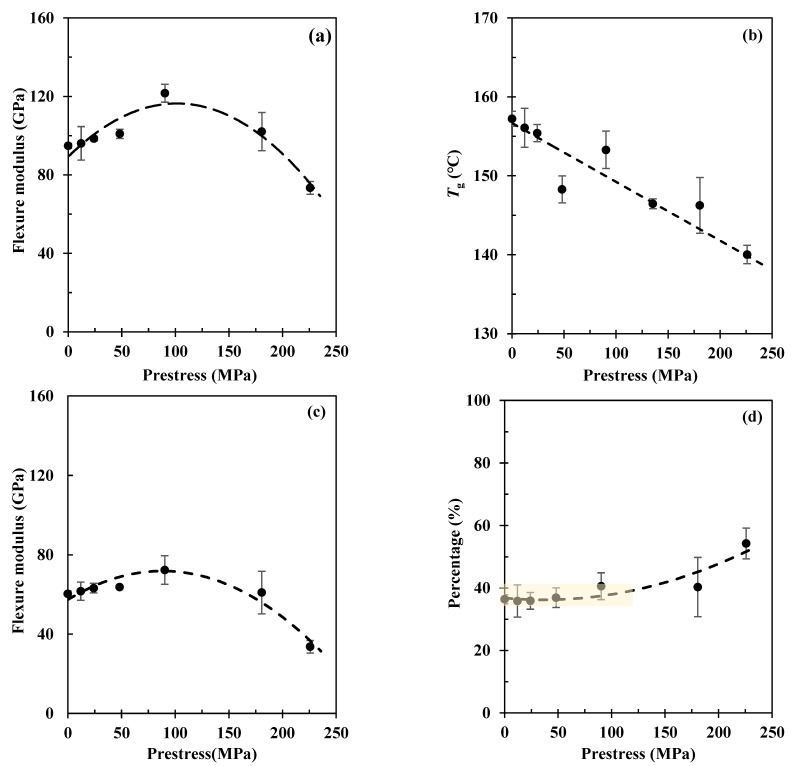
Effects of fibre prestressing level on (**a**) flexure modulus at 30 °C (glassy region), (**b**) glass transition temperature, *T*_g_, (**c**) flexure modulus at 180 °C (rubbery plateau region), and (**d**) contribution percentage of the thermal residual stress of the composite. Each was tested three times, and standard errors are presented by error bars.

Further details are presented by plotting out the effects of prestress level on the flexure modulus at 30 °C (glassy region), see Figure 9a; the glassy temperature, *T*_g_, Figure 9b; and the flexure modulus at 180 °C (rubbery plateau region), as in Figure 9c, respectively. Because beyond *T*_g_, most of the molecular chain segments are considered movable, the thermal residual stress would be released by these movements. Thus, technically, Figure 9a shows the overall effects of internal stress, i.e., $\sigma_{3}\left(t\right)$ as in Figure 1, on the flexure modulus of the composite, whilst Figure 9c presents pure effects from compressive stress generated by the elastic fibre recovery, i.e., $\sigma_{\mathrm{com}}\left(t\right)$ in Figure 1. As for *T*_g_, Figure 9b shows a negative linear reduction with prestress level, referring to the tensile modulus performance shown in Figure 6. This indicates that the prestress is detrimental to the fibre–matrix interface.

Compared with the non-prestressed control sample, the flexure modulus grows with the prestress level until 90 MPa of creep stress, which gives a maximum improvement of 28.3% in the elastic region, and then it decreases very quickly, indicating an optimal prestress level on the flexure modulus of the composite. In the rubbery plateau region at 180 °C, the prestress effects show a similar trend as with the glassy region and give a maximum improvement of 30.0% in flexure modulus. It is interesting to note that this improvement is identical to the increment in the elastic region at 30 °C.

Because fibre prestressing is applied before resin curing, beyond the optimal prestress level, further increases in the creep stress may lead to premature failure of the fibres. Because all the EPPMC samples are fabricated using the same procedure, the thermal residual stresses generated should be at the same level, i.e., they should have the same effects on the flexure modulus. Thus, pure effects from thermal residual stress can be represented by eliminating the pure prestress effects, i.e., Figure 9c, from the overall stress effects, i.e., Figure 9a, in terms of the flexure modulus changes. Figure 9d further presents the contributing percentage of the thermal residual stress at different prestress levels. Apart from the variations in data points, it shows similar contributions from the thermal residual stress until a prestress level of 90 MPa; further increase in prestress leads to an increase in the contribution percentage. It attributes to that carbon fibre is negative in the thermal expansion coefficient, although applied fibre prestressing may induce geometric changes in the cross-sections, these effects on the thermal residual stress are minor. As demonstrated by the tensile and impact strength data, further increasing the prestress level would lead to internal structural damages or local breakages of fibre filaments that will change the generated thermal residual stresses, thus increasing the contribution percentages as in Figure 9d.

### 4.4. Viscoelastic Creep Performance

**Figure 10:**
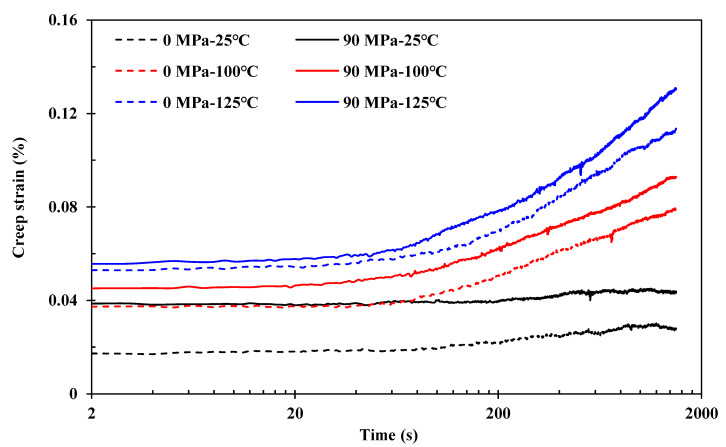
Effects of fibre prestressing level on the viscoelastic creep performance at 25, 100, and 125 °C (below *T*_g_) in logarithmic scale, compared with the non-prestressed control samples.

Figure 10 shows the prestress effects on the viscoelastic creep performance of the composites at temperatures of 25, 100, and 125 °C (below *T*_g_) in a logarithmic scale. The creep strain clearly increases with temperature. Compared with the non-prestressed control samples, the creep strain values increase with applied fibre prestressing under 90 MPa, indicating lower stiffness performance. This is consistent with the performance in terms of modulus in Section 4.1 and glassy temperature in Section 4.3.

### 4.5. Elastic Fibre Prestressing Mechanisms

From both the static and dynamic mechanical analysis shown above, it is clear that fibre prestressing is effective in improving the tensile strength, elongation at break, impact strength, as well as the dynamic flexural modulus, where an optimal prestress level is also observed to maximise the prestress benefits, which is found to be around 90 MPa. The tensile modulus and glassy temperature are found to be negatively proportional to the prestress level, indicating that the interface strength may be influenced by fibre prestressing. The elastic fibre pressing mechanisms thus can be proposed and discussed.

As discussed in Section 2, the internal in-plane stress is dependent on the prestress-generated compressive stresses, i.e., $\sigma_{\mathrm{com}}\left(t\right)$. Although it is time-dependent, the interaction with the viscoelastic matrix would also be stabilised quickly after prestress load removal, which is supported by our short-term DMA testing. Because the internal stress level within a composite plays a vital role in determining the mechanical performance of the composite, the optimal prestress level infers that fibre prestressing is effective in adjusting the internal stress level within an EPPMC. The applied stress thus interacts with the internal stress first, which is then transferred into the fibre and matrix to carry loads until failure.

**Figure 11:**
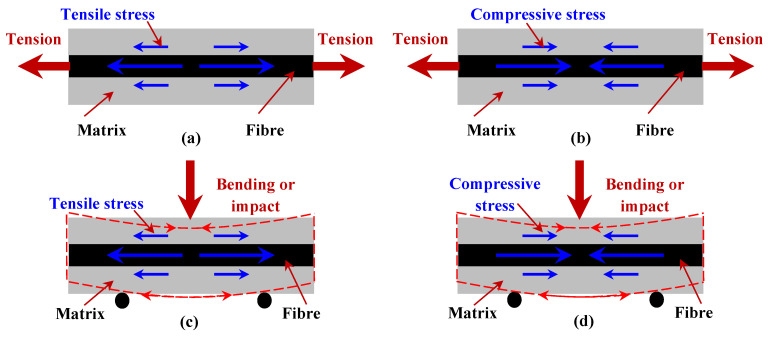
The stress interaction mechanisms within a composite under loading, showing (**a**) as-manufactured composite under tension; (**b**) prestressed composite under tension; (**c**) as-manufactured composite under bending or impact; and (**d**) prestressed composite under bending or impact.

The stress interaction mechanisms are demonstrated in Figure 11 for samples under both tension and compression in terms of without and with fibre prestressing effects. For Figure 11a, an as-manufactured composite would be under tension because the in-plane stress is equal to the thermal residual stress, whereas for a prestressed composite, as in Figure 11b, the compressive stress would interact with the tensile stress and transfer into the fibre and matrix in order to improve the tensile strength, elongation at break, as well as the creep strains. When a composite is under impact or three-point bending, the contacting surface is under compression at the beginning and then under tension until failure, while the outer surface is under tension throughout the loading. For samples without fibre prestressing shown in Figure 11c, stress compensation would only occur on the contacting surface, whilst the outer surface is prone to crack and break under both the thermal residual stress and external tension. Applying fibre prestressing, the internal tensile stress level is counterbalanced by the compressive stresses generated from elastic fibre recovery, see Figure 11d, providing stress compensations on both sample surfaces in order to promote the bending and impact performance.

Although it is demonstrated that there is a constant decrease in terms of the interface bonding strength, the mechanical performance shows that the maximum prestress benefits in terms of flexure modulus and impact strength occur at the same optimal prestress level with the tensile strength, i.e., 90 MPa of creep stress. Further increases in the prestress level will lead to internal structural damages and local breakages of fibre filaments as demonstrated by the contribution percentage changes from the thermal residual stress as discussed in Section 4.3.

**Figure 12:**
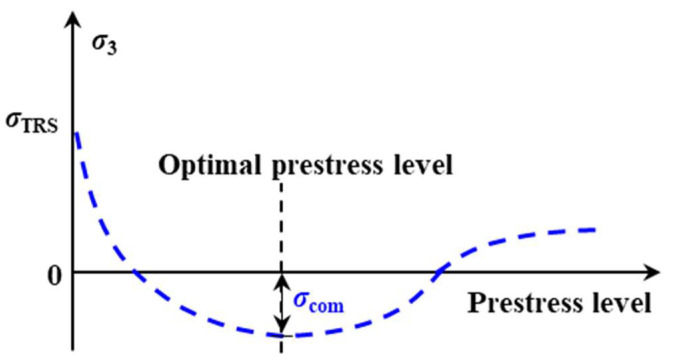
In-plane stress level evolution with prestress level within a composite.

Now we consider the in-plane stress level (i.e., $\sigma_{3}$) evolution within the composite regulated by adjusting the elastic fibre prestressing level, see Figure 12. When the composite is fully cured, the thermal residual stress, *σ*_TRS_, is constant under the same curing process, which is determined using Equation (2) to be 4.20 MPa in tension. By increasing the fibre prestressing level, the constant thermal residual stress interacts with the generated compressive stress, leading to a continuous reduction in in-plane stress. To obtain the optimal prestress benefits in tension, the tensile stress compensations from the compressive stress would also approach the maximum value, corresponding to the maximum compressive in-plane stress as demonstrated in Figure 12. Beyond it, however, internal structural damages and local breakages lead to growth in in-plane stress until prestress-induced fibre failure.

## 5. Conclusions and Future Perspectives

The elastic fibre prestressing (EFP) technique has been developed to balance the thermal residual stress generated during the curing of a polymeric composite. To further understand the EFP mechanics, we established a theoretical model to decouple the EFP principle and performed a comprehensive investigation into the prestress effects in terms of the static and dynamic mechanical properties, as well as the viscoelastic creep performance. The EFP mechanisms are then proposed based on a theoretical and experimental analysis in order to reveal further insight. The main findings include:

(i) The EFP technique is able to be applied to a UD carbon prepreg material through a bespoke fibre prestressing rig to produce prestressed composite samples. It is found that fibre prestressing is effective in destroying the defective fibres before being cured into a matrix, which in turn improves the mechanical strength of the polymeric composite.
(ii) There is an optimal EFP level to maximise the prestress benefits, i.e., 90 MPa for the applied CFRP prepreg. In comparison to the non-prestressed control samples, the tensile strength is improved by 15%, the elongation at break by 11%, the impact strength by 11%, and the dynamic flexure modulus in both the elastic and rubbery plateau regions by ~30%. Beyond the optimal prestress level however, there will be internal structural damages and premature failures which are able to change the in-plane stress levels within the composite.
(iii) The EFP is detrimental to the fibre/matrix interface, both the tensile modulus and creep stiffness of the composite are negatively proportional to the prestress level. This is further supported by the glassy temperature changes after fibre prestressing.
(iv) The established theoretical model indicates further insight and the EFP mechanisms are proposed to reveal the in-plane stress evolution, which is vital to determine the overall performance of the polymeric composite.

Therefore, the EFP technique has been expanded and successfully applied to the CFRP prepreg materials, which is effective to improve the overall static and dynamic mechanical properties without increasing structural weights and dimensions. These are expected to further promote industrial applications of the EFP technique to polymeric composite industries.

## Figures and Tables

**Table 1:** Mechanical properties of a unidirectional carbon prepreg before and after curing.

Type	Specification	
Prepreg	Fibre	Carbon T700
	Thermoset resin	Epoxy 7901
	Gel time	120 °C, 11~17 min
	Resin volume fraction	48%
Laminate	0° Elastic modulus	115 GPa
	0° Tensile strength	2300 MPa
	0° Compressive strength	1050 MPa
	0° Flexural strength	1250 MPa
	0° Interlaminar shear strength	55 MPa

## Data Availability

All the results presented in the manuscript could be requested to the corresponding author.
